# Association between hypertension and osteoporosis: a population-based cross-sectional study

**DOI:** 10.1186/s12891-024-07553-4

**Published:** 2024-06-03

**Authors:** Yuqing Huang, Jianya Ye

**Affiliations:** Department of Orthopedic, Huaian Hospital of Huaian City, No.19, Shanyang Avenue, Huaian District, Huaian, 223200 China

**Keywords:** Hypertension, Osteoporosis, Bone mineral density, Fracture, NHANES

## Abstract

**Background:**

Current evidence suggests that metabolic dysregulation is inextricably linked to both hypertension and osteoporosis, but the correlation between hypertension and osteoporosis is still unclear. Therefore, in this study, we explored the correlation between hypertension and osteoporosis.

**Methods:**

A total of 37,807 participants from the National Health and Nutrition Examination Survey (1999–2010, 2013–2014, 2017–2018) were enrolled in this population-based cross-sectional study. Hypertension was considered an exposure factor and osteoporosis was considered an outcome factor. Logistic regression and subgroup analysis were used to assess the association between hypertension and osteoporosis.

**Results:**

A total of 2,523 participants, with a mean age of 68.65 ± 12.21 years, suffered from osteoporosis, and 86.2% were female. Participants with osteoporosis had a greater prevalence of hypertension than participants without osteoporosis (*p* < 0.001). Participants with hypertension also had a greater prevalence of osteoporosis than participants without hypertension (*p* < 0.001). Univariate logistic regression analysis indicated that hypertension was associated with osteoporosis (OR: 2.693, 95% CI: 2.480–2.924, *p* < 0.001). Multivariate logistic regression analysis with a fully adjusted model indicated that hypertension was strongly associated with osteoporosis (OR: 1.183, 95% CI: 1.055–1.327, *p* = 0.004). Subgroup analysis revealed that the associations between hypertension and osteoporosis were significant in the younger than 60 years, male sex, diabetes subgroup and hypercholesterolemia subgroup (*p* < 0.05).

**Conclusion:**

Hypertension was independently associated with osteoporosis in the general population.

## Introduction

Currently, osteoporosis has become one of the major public health problems, and its prevalence rate is increasing with the aging of the world population [1]. Osteoporosis, which is a bone disease characterised by reduced bone density and increased risk of fracture, constantly threatens the life and health of postmenopausal women and elderly individuals [2]. Wright et al. assessed participants in the 2005–2008 National Health and Nutrition Examination Survey (NHANES) using the new National Bone Health Alliance (NBHA) diagnostic criteria and reported that up to 16% of men and 29.9% of women over the age of 50 were suffering from osteoporosis, with the prevalence in males increasing with age, potentially increasing to 46.3% for men and 77.1% for women [3]. A 2023 Global Burden of Disease (GBD) study reported that the incidence of osteoporosis has more than tripled in the past 30 years, with the global incidence of osteoporosis increasing to 41.5 million in 2019, and that the number of individuals affected by osteoporosis is expected to reach 109 million and 154 million among men and women, respectively, between 2030 and 2034 [4]. A recent GBD study also showed that the number of global deaths caused by osteoporosis and low bone mass has increased by 111.16% over the past 30 years [5]. Within this burden of disease, the risk of fracture due to osteoporosis is substantial and fatal. Bonafede et al. assessed the risk of fracture in US patients over the age of 50 years with osteoporosis and reported that the incidence of hip fracture accounted for 25.4% of all fractures [6]. In addition, Tanha et al. analysed osteoporosis patients over 50 years of age in a single country and reported that the estimated annual incidence rates of hip fracture were 138.26 per 100,000 for men and 157.52 per 100,000 for women [7]. Additionally, the GBD study conducted by Feng et al. revealed a significant increase in the disease burden of hip fractures in patients over the age of 55 years from 1990 to 2019, with the incidence, morbidity and mortality of hip fractures reaching 1191.39, 681.35 and 130.78 per 100,000 people in 2019, respectively, and the disease burden of women was greater than that of men [8]. It has been reported that secondary prevention interventions for osteoporotic fractures can reduce the number of fractures by approximately 5% within 5 years and reduce substantial medical costs [9]. Therefore, screening controllable risk factors in people at high risk of osteoporosis is crucial to reduce the socioeconomic and health burden of osteoporosis.

Current evidence reveals numerous risk factors for osteoporosis, including age, sex hormones, glucocorticoids, suboptimal physical activity, and low intake of vitamin D and calcium [10]. However, with the aging of the population and the dramatic shift in lifestyle, a number of metabolism-related diseases, such as hypertension, are also emerging as risk factors for osteoporosis. Hypertension is also a currently recognised public health problem, has been shown to be an independent risk factor for cardiovascular disease (CVD) and is associated with a poor prognosis for those with CVD. Previous studies have indicated a correlation between low bone mineral density (BMD) and fractures and the prevalence of CVD, and various metabolic risk factors (such as secondary hyperparathyroidism, increased sympathetic outflow, oxidative stress, inhibition of vitamin K-dependent matrix proteins, osteopontin and angiotensin II) or related mechanisms (such as histone modification) have been identified as potentially contributing to the development of arterial stiffness or hypertension and bone metabolism disorders or osteoporosis [11–17]. Therefore, we hypothesized that there was a close correlation between hypertension and osteoporosis. Epidemiologically, a cross-sectional study revealed that hypertension was associated with low levels of 25-hydroxy vitamin D and osteocalcin, suggesting that the low bone turnover caused by hypertension could be one of the mechanisms underlying hypertension-related osteoporosis [18]. Recently, a large cross-sectional study involving 4,306 participants from the 2005–2010 NHANES also revealed that hypertension was associated with higher BMD at the lumbar spine in postmenopausal women and men over 50 years old, but not with BMD at the femoral neck, and lumbar BMD was positively correlated with systolic blood pressure (SBP) and negatively correlated with diastolic blood pressure (DBP) in both sexes [19]. Furthermore, an earlier study involving 2,738 female participants aged 50 and over from the Third NHANES did not find a correlation between hypertension and proximal femur BMD [20]. These data indicate that the relationship between hypertension and BMD remains controversial, and the correlation between hypertension and osteoporosis among participants in the NHANES has not been fully explored. Therefore, based on this research background, we explored the correlation between hypertension and the prevalence of osteoporosis in NHANES participants.

## Methods

### Study population

**Fig. 1:**
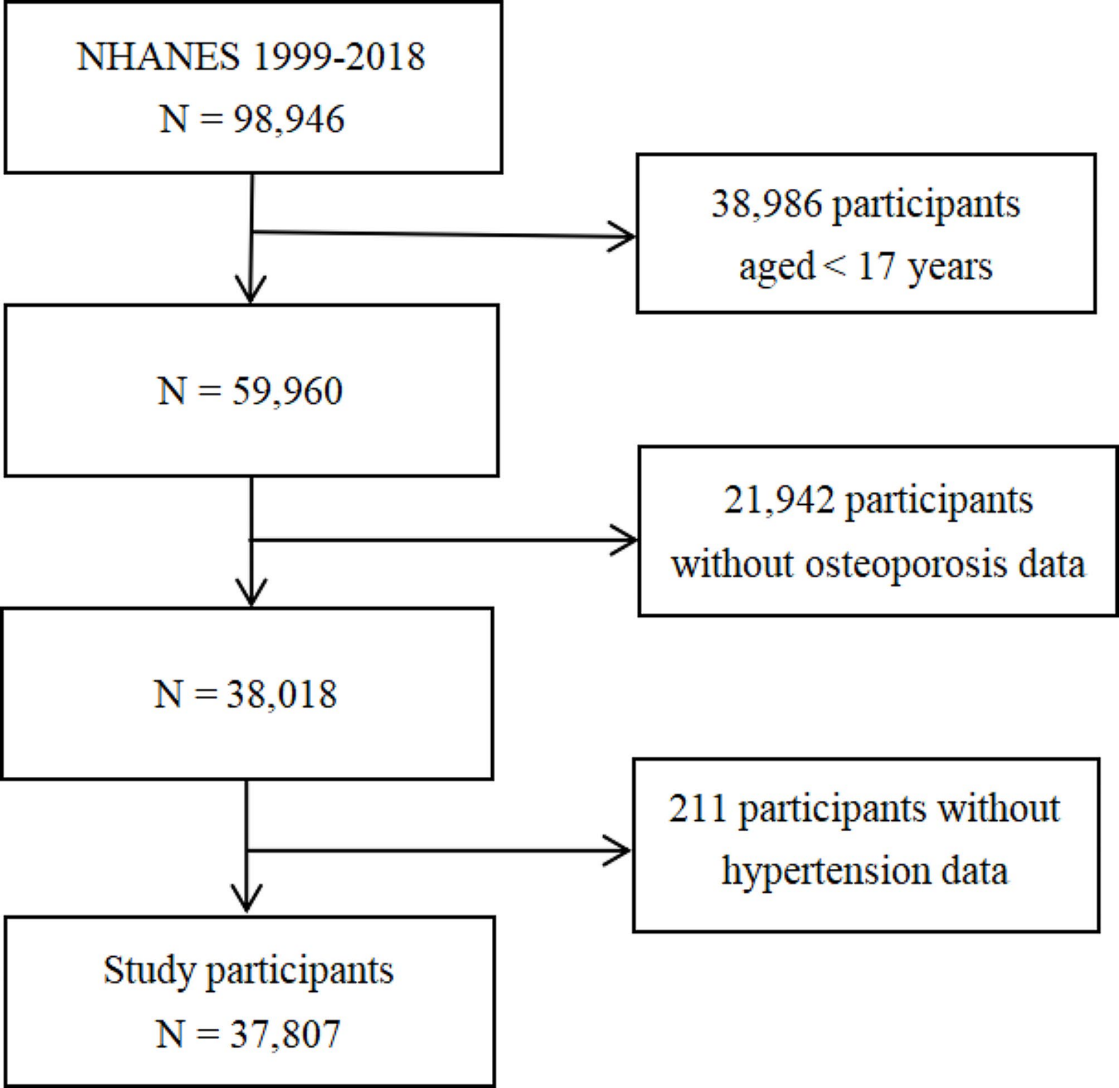
Flowchart of study participants

The NHANES is a multiple-sample survey of the entire U.S. population that began in the early 1960s to assess the health and nutritional status of adults and children in the United States. Beginning in 1999, the NHANES was conducted biennially, focusing on demographic, socioeconomic, dietary, and health conditions, and the data obtained from the survey were used to determine the prevalence of and risk factors for major diseases and to help formulate social and public health policies appropriate for contemporary conditions. For more detailed information, please refer to the official NHANES website homepage (https://www.cdc.gov/nchs/nhanes/). After excluding individuals without data on osteoporosis and hypertension, 37,807 individuals from the NHANES (1999–2010, 2013–2014, 2017–2018) were ultimately included in this cross-sectional observational study (Fig. 1). The National Center for Health Statistics of the Center for Disease Control and Prevention Institutional Review Board approved the protocol. The research program complied with the basic elements of the Declaration of Helsinki. All participants signed an informed consent form while participating in the NHANES.

### Variables and definitions

All data for this study were downloaded from the publicly available NHANES website (https://www.cdc.gov/nchs/nhanes/), including demographic information, comorbidity and medication information obtained from the family interview questionnaire, and biomarker information. Participants were divided into four groups based on their racial attributes: non-Hispanic White, non-Hispanic Black, Mexican-American and others. Participants were categorised into three groups based on the family poverty income ratio (PIR): ≤ 1.0, 1.0–3.0, and > 3.0. Participants were categorised into three groups based on smoking status: every day, some days, and not at all. Diabetes was defined as fasting plasma glucose (FPG) ≥ 7.0 mmol/L, hemoglobin A1c (HbA1c) ≥ 6.5%, or ever having been diagnosed with diabetes by a physician [21]. Hypertension was defined as SBP or DBP ≥ 140 or 90 mmHg, or a previous diagnosis of hypertension by a physician, where the values of SBP and DBP were the average of three non-same-day measurements [22]. Hypercholesterolemia was defined as ever having been diagnosed with hypercholesterolemia by a physician. Osteoporosis was defined as a self-reported history of osteoporosis or the use of anti-osteoporosis drugs. Body mass index (BMI) was measured based on height and weight, that is, BMI = weight (kg)/height (m^2^). Blood markers, including triglyceride, total cholesterol, low-density lipoprotein cholesterol (LDL-C), high-density lipoprotein cholesterol (HDL-C), blood urea nitrogen (BUN), creatinine, albumin, uric acid, FPG, HbA1c, C-reactive protein (CRP), alkaline phosphatase (ALP) and total calcium, were obtained by trained professionals who collected blood specimens from the veins of participants according to standard procedures and sent them to the standard laboratory for measurement.

### Statistical analysis

When performing descriptive statistics, categorical variables were presented as frequencies and percentages and differences between groups were tested with chi-square tests or Fisher’s exact tests. Normally distributed continuous variables were presented as the means and standard deviations and were tested for differences between groups using independent sample t tests, while nonnormally distributed continuous variables were presented as medians and quartiles and were tested for differences between groups using Mann‒Whitney U tests. Univariate logistic regression analyses were used to test the associations of all variables with osteoporosis. Variables with *P* < 0.05 were included in multivariate logistic regression analyses and four multivariate adjusted models were constructed, with Model 1 unadjusted for covariates; Model 2 adjusted only for age and sex; Model 3 further adjusted for race, PIR, smoking, hypotensive drugs, diabetes, hypoglycemic drugs, hypercholesterolemia and cholesterol-lowering drugs; and Model 4 adjusted even further for BMI, SBP, DBP, total cholesterol, HDL-C, HbA1c, CRP, albumin, ALP, uric acid, BUN and total calcium. Then multivariate logistic regression models based on Model 4 were used to test the association between hypertension and osteoporosis in multiple subgroups. SPSS 26.0 was used to test all the statistical data, and a two-tailed *p* < 0.05 was considered to indicate statistical significance.

## Results

### Baseline characteristics

As shown in Table 1, compared to those in the non-osteoporosis group, the osteoporosis group was more likely to be female; have a non-Hispanic White status; have a family PIR probability of 1.0–3.0; have never smoked; have a history of hypertension, diabetes, and hypercholesterolemia; be used hypotensive drugs, hypoglycemic drugs, and cholesterol-lowering drugs; have higher SBP, triglyceride, total cholesterol, HDL-C, BUN, FPG, HbA1c, CRP, ALP and total calcium values; and have lower BMI, DBP, albumin and uric acid values (*p* < 0.05). As shown in Table 2, compared with those in the non-hypertension group, the hypertension group was more likely to be female; have a non-Hispanic Black race; have a 1.0–3.0 probability of having family PIR; be never smokers; have a history of osteoporosis, diabetes, and hypercholesterolemia; use hypotensive drugs, hypoglycemic drugs, and cholesterol-lowering drugs; have higher BMI, SBP, DBP, BUN, creatinine, uric acid, FPG, HbA1c, CRP, ALP and total calcium; and have lower total cholesterol, LDL-C, HDL-C and albumin levels (*p* < 0.05).

**Table 1:** Baseline characteristics of participants stratified by the osteoporosis

	All participants	Non-osteoporosis	Osteoporosis	*P* value
Age, years	52.18 ± 18.32	51.01 ± 18.12	68.65 ± 12.21	< 0.001
Sex, male, n (%)	18,094 (47.90%)	17,745 (50.30%)	349 (13.80%)	< 0.001
Race, n (%)				< 0.001
Non-Hispanic White	18,201 (48.10%)	16,602 (47.10%)	1599 (63.40%)	
Non-Hispanic Black	7654 (20.20%)	7363 (20.90%)	291 (11.50%)	
Mexican-American	7047 (18.60%)	6733 (19.10%)	314 (12.40%)	
Others	4905 (13.00%)	4586 (13.00%)	319 (12.60%)	
Family PIR, n (%)				< 0.001
≤ 1.0	6687 (19.50%)	6255 (19.60%)	432 (19.10%)	
1.0–3.0	14,462 (42.20%)	13,374 (41.80%)	1088 (48.10%)	
> 3.0	13,099 (38.20%)	12,355 (38.60%)	744 (32.90%)	
Smoking status, n (%)				< 0.001
Every day	6778 (37.40%)	6449 (38.00%)	329 (28.40%)	
Some days	1509 (8.30%)	1458 (8.60%)	51 (4.40%)	
Not at all	9859 (54.30%)	9079 (53.40%)	780 (67.20%)	
Hypertension, n (%)	13,869 (36.70%)	12,374 (35.10%)	1495 (59.30%)	< 0.001
Diabetes, n (%)	4792 (12.70%)	4337 (12.30%)	455 (18.00%)	< 0.001
Hypercholesterolemia, n (%)	12,055 (43.20%)	10,741 (42.00%)	1314 (56.60%)	< 0.001
Hypotensive drugs, n (%)	10,682 (29.70%)	9393 (28.00%)	1289 (52.80%)	< 0.001
Hypoglycemic drugs, n (%)	4342 (11.50%)	3920 (11.10%)	422 (16.70%)	< 0.001
Cholesterol-lowering drugs, n (%)	6634 (28.00%)	5745 (26.50%)	889 (43.80%)	< 0.001
Body mass index, kg/m^2^	28.84 ± 6.61	28.89 ± 6.61	28.12 ± 6.54	< 0.001
Systolic blood pressure, mmHg	126.50 ± 20.34	125.90 ± 20.04	135.02 ± 22.60	< 0.001
Diastolic blood pressure, mmHg	70.69 ± 12.29	70.87 ± 12.26	68.17 ± 12.42	< 0.001
Triglyceride, mmol/L	1.28 (0.89, 1.89)	1.31 (0.90, 1.93)	1.40 (1.02, 2.09)	0.041
Total cholesterol, mmol/L	5.14 ± 1.11	5.13 ± 1.12	5.21 ± 1.10	0.002
Low-density lipoprotein cholesterol, mmol/L	3.02 ± 0.94	3.03 ± 0.94	2.98 ± 0.93	0.125
High-density lipoprotein cholesterol, mmol/L	1.37 ± 0.42	1.36 ± 0.42	1.52 ± 0.46	< 0.001
Blood urea nitrogen, mmol/L	4.95 ± 2.27	4.90 ± 2.24	5.72 ± 2.49	< 0.001
Creatinine, umol/L	74.26 (61.88, 88.40)	72.49 (61.88, 88.40)	70.72 (61.88, 88.40)	0.074
Albumin, g/L	42.08 ± 3.68	42.14 ± 3.70	41.22 ± 3.33	< 0.001
Uric acid, umol/L	323.33 ± 87.84	324.19 ± 87.76	310.95 ± 88.00	< 0.001
Fasting plasma glucose, mmol/L	5.50 (5.10, 6.11)	5.50 (5.08, 6.11)	5.63 (5.16, 6.31)	< 0.001
Hemoglobin A1c, %	5.72 ± 1.08	5.71 ± 1.09	5.84 ± 0.96	< 0.001
C-reactive protein, mg/dL	0.22 (0.09, 0.51)	0.22 (0.09, 0.51)	0.25 (0.11, 0.56)	< 0.001
Alkaline phosphatase, U/L	69.00 (56.00, 85.00)	69.00 (56.00, 85.00)	71.00 (57.00, 88.50)	0.003
Total calcium, mmol/L	2.36 ± 0.10	2.36 ± 0.10	2.37 ± 0.11	< 0.001

Data were expressed as mean ± SD, median (first quartile, third quartile), or n (%). PIR, poverty income ratio

**Table 2:** Baseline characteristics of participants stratified by the hypertension

	Non-hypertension	Hypertension	*P* value
Age, years	46.44 ± 17.77	62.10 ± 14.66	< 0.001
Sex, male, n (%)	11,617 (48.50%)	6477 (46.70%)	0.001
Race, n (%)			< 0.001
Non-Hispanic White	11,465 (47.90%)	6736 (48.60%)	
Non-Hispanic Black	4101 (17.10%)	3553 (25.60%)	
Mexican-American	5104 (21.30%)	1943 (14.00%)	
Others	3268 (13.70%)	1637 (11.80%)	
Family PIR, n (%)			< 0.001
≤ 1.0	4261 (19.60%)	2426 (19.40%)	
1.0–3.0	8838 (40.70%)	5624 (44.90%)	
> 3.0	8618 (39.70%)	4481 (35.80%)	
Smoking status, n (%)			< 0.001
Every day	4654 (42.10%)	2124 (30.00%)	
Some days	1073 (9.70%)	436 (6.20%)	
Not at all	5336 (48.20%)	4523 (63.90%)	
Osteoporosis, n (%)	1028 (4.30%)	1495 (10.80%)	< 0.001
Diabetes, n (%)	1470 (6.10%)	3322 (24.00%)	< 0.001
Hypercholesterolemia, n (%)	5072 (32.60%)	6983 (56.60%)	< 0.001
Hypotensive drugs, n (%)	639 (33.80%)	216 (35.10%)	< 0.001
Hypoglycemic drugs, n (%)	1289 (5.40%)	3053 (22.00%)	< 0.001
Cholesterol-lowering drugs, n (%)	1954 (15.00%)	4680 (43.70%)	< 0.001
Body mass index, kg/m^2^	27.76 ± 6.05	30.72 ± 7.11	< 0.001
Systolic blood pressure, mmHg	120.87 ± 17.24	136.07 ± 21.59	< 0.001
Diastolic blood pressure, mmHg	69.91 ± 11.29	72.08 ± 13.76	< 0.001
Triglyceride, mmol/L	1.25 (0.87, 1.85)	1.46 (1.03, 2.11)	< 0.001
Total cholesterol, mmol/L	5.16 ± 1.10	5.10 ± 1.14	< 0.001
Low-density lipoprotein cholesterol, mmol/L	3.07 ± 0.94	2.93 ± 0.94	< 0.001
High-density lipoprotein cholesterol, mmol/L	1.39 ± 0.42	1.35 ± 0.42	< 0.001
Blood urea nitrogen, mmol/L	4.51 ± 1.73	5.72 ± 2.82	< 0.001
Creatinine, umol/L	70.72 (61.88, 84.86)	79.56 (64.53, 96.36)	< 0.001
Albumin, g/L	42.41 ± 3.76	41.52 ± 3.48	< 0.001
Uric acid, umol/L	308.25 ± 81.56	349.64 ± 92.11	< 0.001
Fasting plasma glucose, mmol/L	5.38 (5.00, 5.83)	5.83 (5.33, 6.77)	< 0.001
Hemoglobin A1c, %	5.54 ± 0.94	6.04 ± 1.24	< 0.001
C-reactive protein, mg/dL	0.20 (0.08, 0.46)	0.27 (0.12, 0.64)	< 0.001
Alkaline phosphatase, U/L	67.00 (55.00, 83.00)	72.00 (59.00, 88.00)	< 0.001
Total calcium, mmol/L	2.35 ± 0.09	2.36 ± 0.10	< 0.001

Data were expressed as mean ± SD, median (first quartile, third quartile), or n (%). PIR, poverty income ratio

### Logistic regression analysis of hypertension and osteoporosis

As shown in Table 3, univariate logistic regression analysis revealed a significant association between age, sex, race, family PIR, smoking status, hypertension status, diabetes status, hypercholesterolemia status, use of hypotensive drugs, use of hypoglycemic drugs, use of cholesterol-lowering drugs, BMI, SBP, DBP, total cholesterol, HDL-C, BUN, albumin, uric acid, HbA1c, CRP, ALP, and total calcium and the prevalence of osteoporosis (*p* < 0.05), and the prevalence of osteoporosis was 2.69 times greater in participants with hypertension than in those without hypertension (OR: 2.693, 95% CI: 2.480–2.924, *p* < 0.001).

**Table 3:** Univariate logistic regression analysis of osteoporosis

	OR	95% CI	*P* value
Age, years	1.067	1.064–1.070	< 0.001
Sex, male	0.159	0.141–0.178	< 0.001
Race			< 0.001
Non-Hispanic White	Ref	Ref	
Non-Hispanic Black	0.410	0.361–0.466	
Mexican-American	0.484	0.428–0.548	
Others	0.722	0.638–0.818	
Family PIR			< 0.001
≤ 1.0	Ref	Ref	
1.0–3.0	1.178	1.049–1.322	
> 3.0	0.872	0.771–0.985	
Smoking status			< 0.001
Every day	0.594	0.520–0.678	
Some days	0.407	0.305–0.543	
Not at all	Ref	Ref	
Hypertension	2.693	2.480–2.924	< 0.001
Diabetes	1.570	1.411–1.746	< 0.001
Hypercholesterolemia	1.799	1.651–1.960	< 0.001
Hypotensive drugs	2.876	2.647–3.124	< 0.001
Hypoglycemic drugs	1.607	1.440–1.793	< 0.001
Cholesterol-lowering drugs	2.164	1.972–2.374	< 0.001
Body mass index, kg/m^2^	0.981	0.975–0.988	< 0.001
Systolic blood pressure, mmHg	1.019	1.017–1.021	< 0.001
Diastolic blood pressure, mmHg	0.982	0.979–0.986	< 0.001
Total cholesterol, mmol/L	1.061	1.022–1.102	0.002
High-density lipoprotein cholesterol, mmol/L	2.156	1.970–2.360	< 0.001
Blood urea nitrogen, mmol/L	1.123	1.107–1.139	< 0.001
Albumin, g/L	0.938	0.928–0.948	< 0.001
Uric acid, umol/L	0.998	0.998–0.999	< 0.001
Hemoglobin A1c, %	1.099	1.063–1.137	< 0.001
C-reactive protein, mg/dL	1.064	1.018–1.113	0.006
Alkaline phosphatase, U/L	1.002	1.001–1.004	< 0.001
Total calcium, mmol/L	2.494	1.603–3.880	< 0.001

PIR, poverty income ratio; OR, odd ratio; CI, confidence interval

As shown in Table 4, multivariate logistic regression analysis indicated that when adjusting only for age and sex, the prevalence of osteoporosis increased by 23.4% in individuals with hypertension compared with individuals without hypertension (OR: 1.234, 95% CI: 1.127–1.351, *p* < 0.001), while this prevalence increased by 53.8% when further adjusting for race, family PIR, smoking status, hypotensive drugs, diabetes status, hypoglycemic drugs, hypercholesterolemia and cholesterol-lowering drugs (OR: 1.538, 95% CI: 1.240–1.906, *p* < 0.001) and decreased to 18.3% when adjusting even further for BMI, SBP, DBP, total cholesterol, HDL-C, HbA1c, CRP, albumin, ALP, uric acid, BUN and total calcium (OR: 1.183, 95% CI: 1.055–1.327, *p* = 0.004).

**Table 4:** Association of hypertension with osteoporosis

	Non-hypertension	Hypertension	
	OR (95% CI)	OR (95% CI)	*P* value
Model 1	Ref	2.693 (2.480, 2.924)	< 0.001
Model 2	Ref	1.234 (1.127, 1.351)	< 0.001
Model 3	Ref	1.538 (1.240, 1.906)	< 0.001
Model 4	Ref	1.183 (1.055, 1.327)	0.004

Model 1: unadjusted; Model 2: adjusted for age and sex; Model 3: adjusted for variables included in Model 2 and race, PIR, smoking, hypotensive drugs, diabetes, hypoglycemic drugs, hypercholesterolemia and cholesterol-lowering drugs; Model 4: adjusted for variables included in Model 3 and body mass index, systolic blood pressure, diastolic blood pressure, total cholesterol, high-density lipoprotein cholesterol, hemoglobin A1c, C-reactive protein, albumin, alkaline phosphatase, uric acid, blood urea nitrogen and total calcium. OR, odd ratio; CI, confidence interval

### Stratified association between hypertension and osteoporosis

**Fig. 2:**
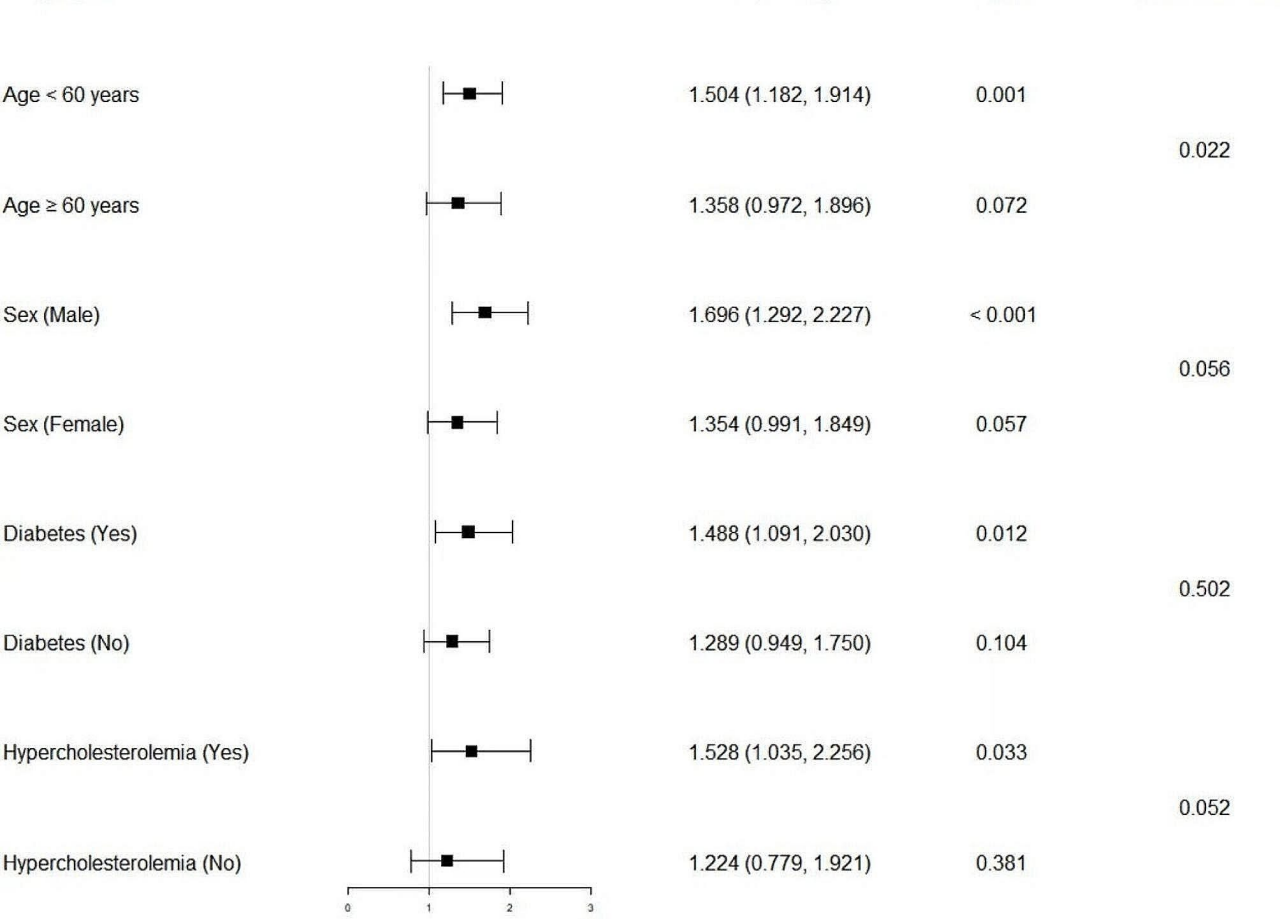
The forest plot for subgroup analysis. The multivariate adjusted model used in the subgroups analysis consisted of all covariates used in model 4 in Table 4 except for the variable that was used for stratification. The interaction of hypertension and variables used for stratification was examined by likelihood ratio tests. OR, odd ratio; CI, confidence interval

As shown in Fig. 2, subgroup analysis indicated that the prevalence of osteoporosis in hypertensive participants was increased by 50.4%, 69.6%, 48.8% and 52.8% in the less than 60 years of age, male, diabetes and hypercholesterolemia subgroups, respectively, than in the nonhypertensive subgroup (OR: 1.504, 95% CI: 1.182–1.914, *p* = 0.001; OR: 1.696, 95% CI: 1.292–2.227, *p* < 0.001; OR: 1.488, 95% CI: 1.091–2.030, *p* = 0.012; OR: 1.528, 95% CI: 1.035–2.256, *p* = 0.033; respectively).

## Discussion

Hypertension and osteoporosis are both currently important public health problems, and there may also be common metabolic pathways between them, but the impact of hypertension on osteoporosis requires further study. In this study, we demonstrated a significant association between the prevalence of hypertension and a greater prevalence of osteoporosis, independent of traditional confounders, in a general population from the United States. In addition, stratified correlations between these variables were confirmed in multiple subgroups. These findings suggest that in patients with hypertension, we should not only screen for prevalence of osteoporosis but also develop corresponding prevention strategies in different populations to reduce the high burden of osteoporosis.

Osteoporosis is a metabolic degenerative bone disease characterised by decreased bone mass, increased bone fragility, structural deterioration of bone tissue, and a high prevalence of fracture and is assessed in clinical practice mainly by BMD [1, 3]. However, this assessment method is time-consuming and expensive, so some researchers have tried to explore novel predictive biomarkers for osteoporosis. For example, in an observational study involving 1,103 participants aged ≥ 50 years, an association between a higher dietary inflammation index and a greater prevalence of osteoporosis was also revealed, but this association was limited to women [23]. In addition, a correlation between heavy metals and osteoporosis was also reported; that is, higher levels of blood cadmium and lower levels of blood selenium were strongly associated with a greater prevalence of osteoporosis in the middle-aged and older US population [24]. Furthermore, lipid metabolism may also be involved in the occurrence and development of osteoporosis. For example, Sun et al. reported that quercetin can reduce osteoporosis caused by testosterone deficiency by inhibiting lipid metabolism [25]. Moreover, another cross-sectional study also found that higher triglyceride and total cholesterol levels were associated with a greater prevalence of osteoporosis [26]. It was also shown that participants with higher apolipoprotein A1 levels had a 71.2% greater risk of developing osteoporosis than those with lower apolipoprotein A1 levels, and for each one-unit increase in apolipoprotein A1, the risk was 1.29 times greater [27]. Moreover, two previous studies demonstrated that the renin-angiotensin system (RAS) plays a significant role in osteoporosis, where the activation of the local RAS in bone tissue can lead to increased bone resorption, thereby triggering osteoporosis, and the use of RAS inhibitors can significantly reduce the incidence of osteoporosis and osteoporotic fractures [28, 29]. Additionally, there is evidence indicating that lower BMD and osteoporotic fractures are significantly associated with a greater incidence of CVD during the follow-up period [15]. These data suggest that hypertension and osteoporosis may share some pathological mechanisms. Several previous studies have confirmed potential common risk factors between hypertension and osteoporosis, such as metabolic factors (secondary hyperparathyroidism, increased sympathetic outflow, oxidative stress, inhibition of vitamin K-dependent matrix proteins, osteopontin, and angiotensin II) and related molecular mechanisms (histone modification) [11–14, 16, 17].Varenna et al. found a covariate correlation between the prevalence of osteoporosis and the prevalence of hypertension in 3,301 postmenopausal women, and that low dairy intake increased the prevalence of both diseases, which suggests that dairy intake may also be a common pathogenic link between hypertension and osteoporosis [30]. Subsequently, in an observational study involving only 518 participants, Hu et al. demonstrated that lower levels of osteocalcin and 25-hydroxy vitamin D were closely associated with a greater prevalence of hypertension, suggesting that abnormalities in bone metabolism may play a role in the development and progression of hypertension [18]. Similarly, after investigating 4,306 individuals from the NHANES, Li et al. found that hypertension was associated with greater lumbar spine BMD in men over 50 years of age and postmenopausal women, but this significant correlation was not detected for femoral neck BMD [19]. Additionally, they further revealed a positive correlation between lumbar BMD and SBP and a negative correlation with DBP, indicating that the relationship between hypertension and BMD is not entirely consistent and may be influenced by individual differences [19]. Additionally, Chai et al. confirmed that the prevalence of osteoporosis was 1.30 times greater in hypertensive patients than in those without hypertension in a cross-sectional study including 2,873 participants from China, but this study included fewer factors, and the multivariate regression model was not adjusted for additional risk factors for osteoporosis, and subgroup analyses were not performed, making the results unrepresentative [31]. Contrary to the above studies, Mussolino et al. conducted a nationally representative cross-sectional cohort study involving 2,738 women aged 50 and above from the Third NHANES, and found that the correlation between hypertension and BMD was no longer significant after adjusting for a greater number of confounding factors [20]. The above data suggest that although there may be some common pathological mechanisms between hypertension and osteoporosis at the molecular level, the correlation between hypertension and osteoporosis at the phenotypic level may still be controversial. Additionally, the exploration of the correlation between hypertension and osteoporosis in certain specific populations remains insufficient. Therefore, based on current research progress, we continued to explore epidemiological evidence of the correlation between hypertension and osteoporosis among participants from the NHANES. Our findings indicate that, after adequately adjusting for confounding factors, individuals with hypertension still had a 1.18 times greater prevalence of developing osteoporosis than did those without hypertension. Moreover, in specific populations such as those under 60 years of age, males, diabetic patients, and those with hypercholesterolemia, the prevalence of osteoporosis in individuals with hypertension could range from 48.8 to 69.6%. However, these studies are just the tip of the iceberg and more basic and clinical studies are needed to explore their correlation.

In addition, the potential mechanisms underlying the link between hypertension and osteoporosis require further discussion. As mentioned above, the RAS can also increase the prevalence of hypertension and osteoporosis, whereas the use of RAS inhibitors can reduce the risk of both hypertension and osteoporosis, which suggests that the RAS may be a common pathophysiological mechanism for hypertension and osteoporosis [28, 29]. Besides, salt intake, dairy intake, and sodium intake status may also be potentially linked, as the intake of these food components as well as antihypertensive drugs has been associated with the prevalence of hypertension and osteoporosis [28–30, 32–34]. Additionally, there is evidence that patients with Cushing’s syndrome exhibit clinical signs of both severe hypertension and osteoporosis, and some underlying pathological mechanisms may contribute to these outcomes [35]. Finally, we also found that the genes encoding fibroblast growth factor and fibroblast growth factor receptor-like 1 may have positive regulatory effects on the prevalence of hypertension and osteoporosis in Korean adults [36]. However, these mechanisms alone are not enough, and there are many more potential mechanisms that need to be explored in future studies to maximise the results of translational medicine.

Although this study confirmed the correlation between hypertension and osteoporosis, there were still several limitations that need to be discussed. First, we did not use BMD to assess the prevalence of osteoporosis in this study due to data limitations, so the prevalence of osteoporosis obtained from the family interview questionnaire may not be representative. Second, the prevalence of hypertension among participants was not assessed in a hospital setting, so further validation is needed to determine whether the prevalence of hypertension from epidemiological sources is sufficiently representative. Third, this study was not adjusted for appropriate weights, so the findings were not representative of the entire U.S. population, but we were sufficient to confirm the correlation between hypertension and osteoporosis in these participants. Fourth, this study did not include dietary, environmental, or genetic factors, which are strongly associated with hypertension and osteoporosis, so there were unavoidable limitations to the results. Finally, we did not validate the stability of the results in postmenopausal women because too much information on women’s menopausal history was missing. In future studies, we hope to remedy these deficiencies. Despite the limitations of this article, there were still some novelties worth discussing: (1) this was based on a population-based cross-sectional study design, revealing the statistical association between hypertension and osteoporosis, providing a new perspective for the joint prevention and treatment of these two diseases; (2) by controlling for various potential confounding factors, this study may propose mechanisms through which hypertension directly or indirectly affects the risk of osteoporosis, providing a scientific basis for future clinical guidance and public health strategy formulation.

## Conclusions

In conclusion, this observational study revealed a strong association between hypertension and a greater prevalence of osteoporosis, suggesting that we should pay attention to the extracardiovascular effects of hypertension and that timely and effective preventive strategies should be developed for people at high risk of osteoporosis to reduce the high prevalence of osteoporosis and the burden of disease.

## Data Availability

The datasets used this study are available from the corresponding author on reasonable request.
